# Long-term outcomes after gonadotropin-releasing hormone agonist treatment in boys with central precocious puberty

**DOI:** 10.1371/journal.pone.0243212

**Published:** 2020-12-10

**Authors:** Young Suk Shim, Kyung In Lim, Hae Sang Lee, Jin Soon Hwang

**Affiliations:** 1 Department of Pediatrics, Hallym University Medical Center, School of Medicine, Hallym University, Seoul, South Korea; 2 Department of Pediatrics, Gachon University School of Medicine, Gil Medical Center, Seoul, South Korea; 3 Department of Pediatrics, Ajou University School of Medicine, Ajou University Hospital, Suwon, South Korea; Universite de Rouen, FRANCE

## Abstract

**Objective:**

Gonadotropin-releasing hormone agonist (GnRHa) treatment improves the potential for gaining height in patients with central precocious puberty (CPP). However, most studies have focused on girls because CPP in boys is relatively rare. Therefore, we aimed to determine the effect of GnRHa treatment on auxological outcomes in boys with CPP.

**Methods:**

Eighty-five boys with CPP were treated with leuprolide or triptorelin acetate 3.75 mg over 2 years. Anthropometry, bone age, sexual maturity rating, and predicted adult height (PAH) were assessed every 6 months. Furthermore, 20 boys were followed up after treatment discontinuation until achievement of the final adult height (FAH).

**Results:**

The mean chronological age (CA) and bone age (BA) of the patients with CPP at treatment initiation were 9.5 ± 0.5 years and 11.7 ± 0.9 years, respectively. The mean duration of treatment was 2.87 ± 0.63 years. The PAH at treatment initiation was 172.1 cm (-0.23 ± 1.05 PAH standard deviation score). The PAH at treatment discontinuation (176.2 ± 6.6 cm) was significantly higher than the pretreatment PAH. In addition, the mean final adult height in the 20 boys who were followed up after discontinuation of treatment was 173.4 ± 5.8 cm, which was significantly higher than the initial PAH (170.1 ± 4.5 cm; *p =* 0.006). In multivariate analysis, the height gain (the difference between the FAH and PAH at treatment initiation) significantly correlated with the target height.

**Conclusion:**

Long-term GnRHa treatment significantly improved the growth potential and FAH in boys with CPP.

## Introduction

Precocious puberty (PP) refers to the development of secondary sexual characteristics before the age of 8 and 9 years in girls and boys, respectively [1]. Central precocious puberty (CPP) is caused by early maturation of the hypothalamic–pituitary–gonadal axis. It is caused by organic brain disorders such as tumors, hemorrhage, or infection in approximately 40%–50% boys with CPP [1]. It can also be idiopathic. The main goal of treatment for CPP is to suppress the gonadal sex steroid secretion effectively to stop premature sexual maturation. Additionally, the treatment aims to preserve the potential to achieve acceptable adult height in each individual based on genetic determinants by suppressing the accelerated skeletal advancement [1].

Currently, gonadotropin-releasing hormone agonists (GnRHa) have been used in the treatment of CPP [1–5]. Although extensive research has been conducted in girls with CPP, very few studies have assessed the long-term outcome of GnRHa treatment in boys with CPP because the incidence of CPP in boys is approximately 10 times lower than that in girls [6–10]. Therefore, this study aimed to determine the effects of GnRHa treatment on auxological outcomes in boys with CPP.

## Subjects and methods

### Patients

Clinical records of 85 boys with CPP who were treated with GnRHa for >2 years at Ajou University Hospital, South Korea from 2007 to 2017 were reviewed. CPP was diagnosed based on the following criteria: (1) objective testicular volume ≥4 mL before 9 years of age, (2) advanced bone age (BA) >1 year above the chronological age (CA), and (3) peak values of pubertal luteinizing hormone (LH) (cutoff value: ≥5 IU/L) achieved during a GnRH stimulation test. Plasma thyroxine and thyroid-stimulating hormone levels were also measured to exclude hypothyroidism. Boys with brain tumor, congenital adrenal hyperplasia, hypothyroidism, or those who received cranial irradiation were excluded from the study. Patients who were treated with growth hormones were also excluded. Of the 85 boys with CPP, 76 boys underwent magnetic resonance imaging (MRI) of the hypothalamic–pituitary area. The other 9 patients refused to undergo MRI. MRI abnormality was not detected in 68 subjects. Brain lesions not definitively related to CPP were found in 8 boys. These included pituitary hyperplasia (n = 4), Rathke’s cleft cyst (n = 2), pineal cyst (n = 1), and arachnoid cyst (n = 1). However, none of the 8 boys had any neurologic symptoms such as headache or seizure.

The standard treatment regimen was subcutaneous administration of leuprolide or triptorelin acetate 3.75 mg every 4 weeks. Twenty-four patients were treated with leuprolide acetate and the other 61 patients were treated with triptorelin acetate. Height, weight, Tanner stage, BA, LH concentration, follicle-stimulating hormone (FSH) concentration, and testosterone concentration were evaluated every 6 months. The LH level was determined 30 min after GnRHa injection every 6 months during the treatment to monitor the suppression of the hypothalamic–pituitary–gonadal axis. LH level <3 IU/L was considered therapeutic suppression [11, 12]. Treatment was discontinued at the bone age of 13 to 13.5 years. Among the 85 boys, 20 boys were followed up after treatment discontinuation until achievement of the final adult height (FAH). We defined FAH as a height velocity of <1 cm/year and bone age >16.5 years [13].

### Methods

Height was measured to the nearest 0.1 cm using a Harpenden stadiometer (Dong Sahn Jenix Co., Ltd., Seoul, South Korea). Weight was recorded to the nearest 0.1 kg using an electronic scale (Cas Co., Ltd., Seoul, South Korea). The volume of each testis was estimated by comparative palpation using a Prader orchidometer by a single pediatric endocrinologist [14]. Pubertal stage was determined by an experienced pediatric endocrinologist according to the method proposed by Marshall and Tanner [15]. Testicular volume was measured using the Prader orchidometer. Target height (TH) was the mean of the parental height plus 6.5 cm. BA was assessed by viewing a radiograph of the left hand and was determined by a single investigator according to the method proposed by Greulich and Pyle [16]. Predicted adult height (PAH) was calculated using the average tables in the Bayley–Pinneau method [17]. Standard deviation scores (SDS) of height, weight, and body mass index (BMI) were calculated using the 2017 growth reference for South Korean children and adolescents, provided by the Korean Pediatric Society and Korea Centers for Disease Control and Prevention [18]. A GnRH stimulation test (Relefact; Sanofi-Aventis, Frankfurt am Main, Germany) was performed. Serum FSH and LH levels were measured at baseline and at 30, 45, 60, and 90 min after administration of 100 μg GnRH. Serum LH and FSH levels were measured using Immunoradiometric Assay (BioSource SA, Nivelles, Belgium). Testosterone levels were determined using a radioimmunoassay, Coat-A-Count (Diagnostic Products, Los Angeles, CA, USA).

### Ethics approval and consent to participate

The protocol was approved by the Institutional Review Board of Ajou University Hospital (AJIRB-MED-OBS-16-372). Written informed consent was obtained from all the subjects or their parents before FAH evaluation.

### Statistical analysis

All statistical analyses were performed using SPSS (ver. 23.0, IBM Corp., Armonk, NY, USA). The values at treatment initiation and at treatment discontinuation were compared using a repeated-measures ANOVA test. Comparisons of the results between the groups were assessed using the independent t-test or Mann–Whitney U test, depending on the data distribution. To determine significant associations with the gain in height (the difference between the FAH and PAH at the initiation of treatment), univariate and multivariate analyses were performed with stepwise variable selection, including age at diagnosis, height SDS, TH, and duration of treatment. Statistical significance was set at *p* < 0.05. Results are presented as mean ± standard deviation, unless indicated otherwise.

## Results

### Patient characteristics

The mean age at diagnosis was 9.5 ± 0.5 years (range: 6.6–9.9 years). The mean duration of GnRHa treatment was 2.87 ± 0.63 years. The mean BA at the time of treatment initiation was 11.7 ± 0.9 years (Table 1). The peak serum LH and FSH levels after GnRH stimulation test were 15.0 ± 9.0 IU/L and 7.5 ± 4.1 IU/L, respectively.

**Table 1:** Clinical characteristics of patients before and after GnRHa treatment (n = 85).

Variable	At treatment initiation	At 1 year of treatment	At treatment discontinuation	*P* value
Age (years)	9.5 ± 0.5	10.7 ± 0.5	12.4 ± 0.7	<0.001
Height (cm)	142.9 ± 5.3	150.4 ± 5.2	159.2 ± 4.9	<0.001
Height SDS	1.25 ± 0.86	1.25 ± 0.81	0.80 ± 0.83	<0.001
Weight SDS	1.28 ± 0.83	1.26 ± 0.82	1.18 ± 0.96	0.087
BMI SDS	1.03 ± 0.95	0.99 ± 0.96	1.08 ± 1.09	0.122
Bone age (years)	11.7 ± 0.9	12.5 ± 0.7	13.5 ± 0.5	<0.001
BA-CA (years)	2.25 ± 0.84	1.85 ± 0.74	1.15 ± 0.68	<0.001
Target height (cm)	171.8 ± 4.1			
Predicted adult height	172.1 ± 5.9	175.0 ± 5.8	176.2 ± 6.6*	<0.001
Testicular volume (cc)	5.0 ± 1.4	4.1 ± 1.1	3.9 ± 1.1	<0.001

* <0.001: compared to the target height

### Effect of GnRHa treatment

The changes in auxological outcomes are summarized in Table 1. After GnRHa treatment, the height SDS decreased significant, while weight and BMI SDS did not change significantly. Moreover, testicular volume decreased significantly during the treatment period. Serum LH levels 30 minutes after GnRHa in all subjects were <3 IU/L during treatment. During the treatment, the growth velocity was 5.71 ± 0.84 cm/year. The rate of growth was 6.27 ± 1.24 cm after 1 year of treatment, after which the growth rate tended to decrease gradually. The mean age and bone age at the time treatment discontinuation were 12.4 ± 0.5 and 13.5 ± 0.5 years, respectively.

There was a significant increase in the PAH after GnRHa treatment in boys with CPP. The PAH at treatment initiation was 172.1 ± 5.9 cm, while that at treatment discontinuation was 176.2 ± 6.6 cm (*p* < 0.001). The PAH at treatment discontinuation was significantly higher than the TH in boys with CPP.

### Final adult height

**Fig 1:**
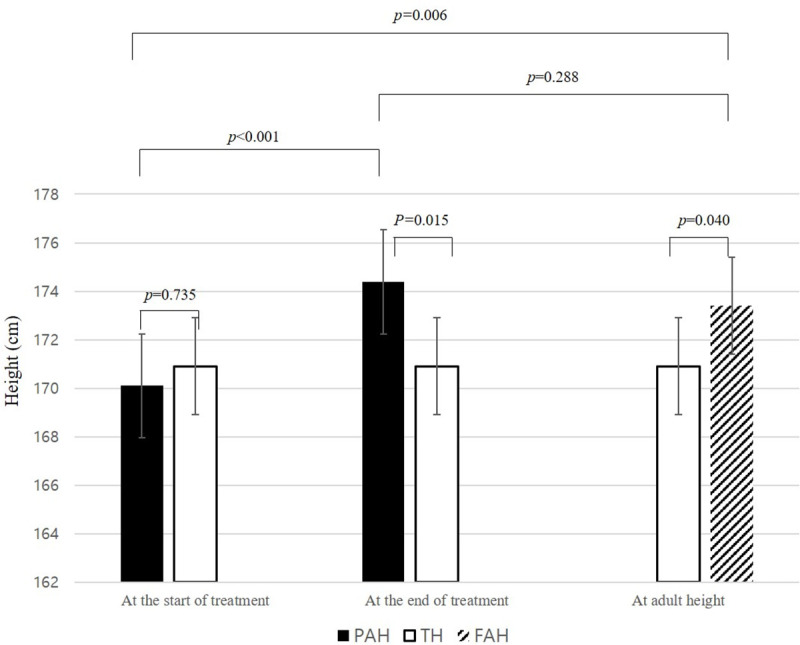
Changes in the PAH during the treatment period and the FAH after GnRHa treatment in 20 boys with central precocious puberty. *PAH, Predicted adult height; TH, target height; FAH, final adult height.

Final auxological data were collected from 20 of 85 boys (Table 2). The final evaluation was performed at a mean age of 15.5 ± 1.4 years after a mean treatment duration of 2.73 ± 0.55 years. The mean final height was 173.4 ± 5.8 cm, and the final height increased significantly compared to the PAH at treatment initiation and the TH. However, there were no statistically significant differences between the FAH and PAH at treatment discontinuation (Fig 1).

**Table 2:** Clinical and auxological characteristics of 20 boys who were followed up until achievement of the final adult height.

	At treatment initiation	At treatment discontinuation	At final adult height
Age (years)	9.2 ± 1.0	12.3 ± 0.6	15.5 ± 1.4
Height (cm)	145.1 ± 5.3	160.1 ± 5.6	173.4 ± 5.8*
Height SDS	1.60 ± 0.91	1.05 ± 0.94	0.01 ± 1.04
Weight SDS	1.47 ± 0.85	1.23 ± 1.04	1.42 ± 1.28
BMI SDS	1.09 ± 0.98	0.98 ± 1.17	1.25 ± 1.33
BA-CA (years)	2.87 ± 0.72	1.58 ± 0.67	
PAH (cm)	170.1 ± 4.7	174.4 ± 4.7	
Target height (cm)	170.9 ± 4.2		

PAH: predicted adult height; BA-CA: the difference between bone age and chronological age

* <0.001: compared to the PAH at treatment initiation and target height

Of the 20 patients, 80% (n = 16) patients achieved a higher FAH than the PAH at treatment initiation. To identify the factors that determined the gain in height, which was defined as the difference between the FAH and PAH at treatment initiation, we divided the 20 boys into two groups—group A, FAH > PAH at treatment initiation (iPAH) (n = 16) and group B, FAH < iPAH (n = 4). We compared clinical variables such as age, height SDS, weight SDS BMI SDS, bone age, and PAH between the two groups. However, the variables were not significantly different between the two groups (data not shown).

### Correlation

In the univariate analysis, the height gain (the difference between the FAH and iPAH) positively correlated with the TH. Moreover, multivariate analysis revealed that the gain in height was influenced significantly only by the TH in 20 boys with CPP who were followed up until achievement of the FAH (Table 3).

**Table 3:** Univariate and multivariate analysis of factors associated with the height gain (the difference between the final adult height and iPAH) in boys treated with gonadotropin-releasing hormone agonist (n = 20, r^2^ = 0.342, *p =* 0.007).

Parameter	Univariate		Multivariate	
	r	*P*	β	*P*
Target height	0.581	0.007	0.585	0.007
Age at treatment initiation	0.052	0.827	0.152	0.448
Height SDS at treatment initiation	0.346	0.135	0.089	0.694
BMI SDS at treatment initiation	0.194	0.413	0.015	0.944
Duration of treatment	0.032	0.892	-0.017	0.934

For stepwise multivariate regression analysis, the following independent variables were entered into the model: age at treatment initiation, height SDS, BMI SDS, target height, and duration of treatment.

## Discussion

In our study population, the PAH significantly increased by approximately 4.1 cm after GnRHa treatment in boys with CPP. The FAH was significantly higher than the iPAH and TH. Moreover, the TH was a strong determinant of the height gain.

Many studies in girls have reported that GnRHa treatment preserves or improves the growth potential in patients with CPP. However, limited data are available on the long-term outcomes of GnRHa treatment on growth in boys with CPP. Previous studies have demonstrated that untreated boys with CPP showed a FAH of approximately -3 standard deviations below the population average [19, 20]. Oerter et al. [21] reported the effect of deslorelin administered subcutaneously at a dose 4 μg/kg/day in 6 boys with CPP for the first time. They observed an improvement in the adult height compared to the pretreatment PAH, although the FAH was significantly lower than the TH by 10 cm. In a study by Paul et al. [20], the near final height in 6 boys treated with various GnRHa regimens was more than the height predicted before therapy. Partsch et al. [22] have suggested that boys with rapid pubertal development who are those likely to achieve below normal height need to be administered GnRHa treatment. Bertelloni and Mull [23] reviewed 11 published articles on the long-term outcomes of GnRHa treatment in 128 boys with CPP. They reported that the mean difference between the iPAH and FAH was -1.4–15.0 cm. Our study also showed that the FAH after GnRHa treatment increased by approximately 3.4 cm compared to the iPAH. However, these studies were conducted in a small number of subjects. Therefore, further studies with larger sample sizes are needed to validate the long-term effect of GnRHa in boys with CPP.

Several factors such as age at treatment initiation, duration of treatment, parental height, and height before treatment influence the growth outcomes after GnRHa treatment in patients with CPP. In our study, the TH was an important predictor, with better long-term growth outcome in 20 boys with CPP. Lazar et al. [24] reported the long-term growth outcomes in 115 girls with CPP and TH, SDS in height, bone age at treatment discontinuation, age at treatment initiation, and SDS of height at the onset of puberty were correlated with the FAH. In another study by Brito et al. [25], the major factors determining the FAH in girls with CPP were TH, SDS of height at treatment initiation and discontinuation, and shorter interval between the onset of puberty and treatment initiation. Oostdijk et al. [26] have reported that height at treatment initiation is the most important positive factor influencing the FAH. Paul et al. [20] have reported that boys with CPP treated before the CA of 5 years achieve a higher gain in height. BA at treatment initiation and discontinuation correlated with the FAH in a previous study in boys with CPP [26]. In another study on CPP, FAH significantly correlated with the duration of treatment, TH, iPAH, and growth velocity during the final year of treatment, while the FAH was inversely correlated with delay in treatment onset, CA at treatment initiation, BA at treatment initiation and discontinuation, and breast stage treatment initiation [27]. Recently, Klein et al. [28] have reported that the rate of change in the BA/CA ratio during GnRHa treatment positively correlated with the PAH in girls and boys with CPP.

Our study has several limitations. First, the number of subjects evaluated for the final height was relatively small. Second, we did not have untreated groups with CPP for ethical reasons. Third, PAH might have been overestimated in this study. Drop et al. [29] reported that the BP method tends to overestimate the FAH in tall boys. However, there are few studies on the validity of prediction methods based on BA in boys with CPP [9, 10]. Lastly, the mean age of patients with CPP in this study was >9 years. However, the determination of age at onset of puberty can be difficult in boys because the exact time of onset of testicular enlargement (≥4 mL) is not as obvious as breast enlargement in girls. Previous studies have reported that a mean delay between the time when the signs of puberty are first observed by the parents and the first consultation with an endocrinologist is 1.5 years [30, 31]. Owing to these difficulties, we included boys who were diagnosed with CPP before 10 years of age [32, 33].

In conclusion, GnRHa treatment can significantly improve the growth potential in boys with CPP. The FAH was significantly higher than the initial height prediction. The target height was the main factor determining the long-term growth outcome.

## Supporting information

S1 File (SAV)
